# Potential drug-drug interactions in patients with indication for prophylactic implantation of a cardioverter defibrillator: a cross-sectional analysis

**DOI:** 10.1186/s12913-020-05131-7

**Published:** 2020-03-31

**Authors:** Christina Dornquast, Mirja Dombrowski, Markus Zabel, Stefan N. Willich, Thomas Reinhold

**Affiliations:** 1 Institute for Social Medicine, Epidemiology and Health Economics, Charité – Universitätsmedizin Berlin, corporate member of Freie Universität Berlin, Humboldt-Universität zu Berlin, and Berlin Institute of Health, Luisenstrasse 57, Berlin, 10117 Germany; 2 Department of Cardiology and Pneumology, Heart Center, University Medical Center, Robert-Koch-Strasse 40, 37075 Göttingen, Germany; 3 DZHK (German Center for Cardiovascular Research), partner site Göttingen, Göttingen, Germany

**Keywords:** Drug-drug interaction, Drug use, Polypharmacy, Prescribing patterns, Implantable defibrillator

## Abstract

**Background:**

Due to demographic transition, multimorbidity and high numbers of medicinal products, polypharmacy rates will presumably further increase. This could lead to higher risks of potentially inappropriate medications with potential drug-drug interactions (PDDI). PDDI has already been investigated by several studies, but not for patients with indication for prophylactic implantation of a cardioverter defibrillator (ICD). Thus, the objective of this analysis was to examine the frequency of PDDI in that specific group of patients and compare patients with or without PDDI regarding potential underlying factors.

**Methods:**

Cross-sectional data analyses were performed using data of the prospective EU-CERT-ICD study that primarily aimed to assess ICD effectiveness in Europe. Self-reported baseline medication data of patients from Germany and Switzerland were used. Patients who reported to take at least two drugs simultaneously for at least 80 days were defined as population at risk. By means of a publicly available interaction checker, we analyzed the medication data regarding occurrence and characteristics of PDDI categorized as minor, moderate, and major PDDI. The analyses were done using descriptive methods and chi square testing.

**Results:**

The total population (*n* = 524) and the population at risk (*n* = 383) were rather similar with an average age of 64 years and about 80% male. PDDIs were found for 296 patients (in 57% of total population vs. 77% of population at risk). The moderate PDDI category was most frequently with 268 affected patients. Comparing patients with and without any PDDI, the proportion of patients with place of residence in Germany varied distinctly (93% vs. 78%). The frequency of any PDDI for the total population was twice as high in Germany as in Switzerland (*p* value < 0.001).

**Conclusions:**

PDDIs were frequently observed in this selected patient population and differed markedly between German and Swiss patients. The results should lead to higher awareness of polypharmacy and PDDIs. Adequate cooperation between health care providers should be promoted and new technologies such as drug interaction information systems or digital patient files used.

**Trial registration:**

The EU-CERT-ICD study is registered at www.clinicaltrials.gov (NCT02064192).

## Background

In the context of demographic transition, associated with a growing proportion of people aged ≥65 years, the increasing frequency of chronic diseases and comorbidities and the growing need of pharmacological treatment, the probability of co-medication up to polypharmacy (defined as five or more concurrent drugs [1]) is high and will presumably continue to rise. An available number of more than 100,000 drugs authorized on the German health care market supports this assumption [2]. The importance of the pharmaceutical market in Germany can also be seen, among other things, in the fact that health expenditure on drugs has almost doubled in the last 20 years and amounted to approximately 52 billion euros in 2017 [3].

Several studies assessed the frequency of drug use and polypharmacy in Germany during the last years. According to data of the German Health Interview and Examination Survey for Adults, nearly three quarters of their participants reported that they have taken at least one drug or food supplement in the last 7 days [4]. A report on drug use for insurants of a large German statutory health insurance company supported these findings [5]. The average number of preparations differed between insured men and women from 2.0 to 3.1. The frequency of a prescribed polypharmacy was 13.6% in women and 9.9% in men [4].

All these mentioned factors of demographic change, multimorbidity, high number of authorized drugs and presumably rising number of polypharmacy leads to higher risks of potentially inappropriate medications (PIMs) [6–8]. According to current studies, the prevalence of PIM for elderly in Germany ranges from 22 to 28% [8–10]. There is also evidence that the occurrence of PIM could be a risk factor for hospitalizations or emergency department visits and higher health care costs [11–14]. Effects of PIMs can be manifold and occur, for example, in the form of adverse drug events (ADE) or potential undesired drug-drug interactions (PDDI) [15]. Since there are only few studies in Germany that assessed the frequency of PDDI so far [16], the present analysis investigates the topic of these PDDI. The study is focused on PDDI in patients with indication for prophylactic implantation of a cardioverter defibrillator (ICD), which were included in the EU-CERT-ICD study [17]. The objective was to examine the frequency of PDDI in this specific group of patients descriptively and to compare those patients with or without PDDI to identify potential underlying factors.

## Methods

### Study design and population

The present analysis was based on data collected during the baseline assessment within the prospective EU-CERT-ICD study (Comparative Effectiveness Research to Assess the Use of Primary Prophylactic Implantable Cardioverter Defibrillators in Europe). The EU-CERT-ICD study primarily aimed to generate contemporary clinical outcome data on ICD effectiveness in Europe. The study was conducted between May 2014 and September 2018. Patients aged ≥18 years with an ischemic or non-ischemic (dilated) cardiomyopathy and recommendation for primary prophylactic ICD treatment were included. Patients undergoing ICD treatment were followed as part of the ICD group, while patients who had not received an ICD were part of the control group. The baseline assessment for patients scheduled for an ICD was done prior to the implantation. A detailed description of the study design and methodology of EU-CERT-ICD is reported elsewhere [17] as well as the primary results [18]. By means of the self-reported baseline medication data of the EU-CERT-ICD patients recruited in Germany and Switzerland (only in these countries the drug consumption was detailed documented by the patients itself), we performed cross-sectional analyses about PDDI. Baseline data were used in order to reflect the daily care routine before the study intervention would have any influence on further treatment.

### Assessment of potential drug-drug interactions

The use of regularly taken drugs prior to the onset of the EU-CERT-ICD study was assessed by patients’ itself and documented in a questionnaire covering the drug consumption during the last 3 months (name of the drug, days of intake). For these patient reported drugs, the principal active agent was attached. PDDI can only occur when several drugs are taken concurrently. Therefore, a selection of those patients was made in whom at least two drugs simultaneously have been taken for at least 80 days (during the last 3 months) were documented. On the one hand, this limit of minimum 80 days should ensure that the medication was taken daily and that a tolerance range for forgetting once was considered. On the other hand, drugs that have been recently discontinued were still included in the analysis. These patients were defined as the population at risk.

The occurrence and the kind of PDDI was determined using the publicly available interaction checker provided by drugs.com (underlying database: Cerner Multum drug, herbal and nutraceutical database) [19]. For every patient of the population at risk, all active substances were entered into the interaction database of drugs.com. According to the classification pattern provided by drugs.com, the PDDI found were divided into three categories [19]:

- Minor: Minimally clinically significant. Minimize risk; assess risk and consider an alternative drug, take steps to circumvent the interaction risk and/or institute a monitoring plan.
- Moderate: Moderately clinically significant. Usually avoid combinations; use it only under special circumstances.
- Major: Highly clinically significant. Avoid combinations; the risk of the interaction outweighs the benefit.

Substances for which no equivalent was found were not included in the analysis. In case of multiple naming of an active substance, this was only taken into account once when determining the PDDI.

To characterize the study population, baseline information about age, sex, place of residence, race and most frequent potential comorbidities were considered.

### Statistical analysis

All analyses were considered explorative. Characteristics of patients, frequency of PDDI categories as well as the comparison of patients with or without PDDI were analyzed using descriptive methods of means and standard deviations for continuous data and absolute and relative frequencies for categorical data. We performed chi square testing for those categorical variables where the comparison of patients with or without PDDI showed relevant descriptive differences. All analyses were done using SPSS for Windows (IBM SPSS Statistics 24).

## Results

### Characteristics of study population

The characteristics of the study population are presented in Table 1 separately for the total population (*n* = 524) and the subsample of the population at risk (*n* = 383) taking ≥2 drugs daily.

**Table 1:** Characteristics of all patients and patients with ≥2 drugs

		All patients (*n* = 524)		Patients with ≥ 2 drugs (*n* = 383)	
		n (%) / Mean ± SD		n (%) / Mean ± SD	
Age (in years)		64.4	12.6	64.3	12.5
Sex	*Male*	411	(78.4)	304	(79.4)
	*Female*	113	(21.6)	79	(20.6)
Place of residence	*Germany*	453	(86.5)	348	(90.9)
	*Switzerland*	71	(13.5)	35	(9.1)
Race	*White*	501	(95.6)	369	(96.3)
	*Black*	4	(0.8)	3	(0.8)
	*Asian*	6	(1.1)	4	(1.0)
	*Unknown*	13	(2.5)	7	(1.8)
Number of comorbidities	*0*	108	(20.6)	81	(21.1)
	*1*	194	(37.0)	143	(37.3)
	*2*	143	(27.3)	101	(26.4)
	*3*	66	(12.6)	46	(12.0)
	*4*	11	(2.1)	10	(2.6)
	*5*	2	(0.4)	2	(0.5)
Most frequent comorbidities	*Hypertension*	359	(68.5)	260	(67.9)
	*Diabetes*	156	(29.8)	121	(31.6)
	*Stroke/TIA*	59	(11.3)	44	(11.5)
	*COPD*	53	(10.1)	39	(10.2)
	*Peripheral arterial disease*	45	(8.6)	28	(7.3)
Number of taken drugs		3.6	2.6	4.7	2.2

*SD* Standard deviation, *TIA* Transient ischaemic attack, *COPD* Chronic obstructive pulmonary disease

In both populations, the mean age of the patients was 64 years and about 80% were male. The most frequent comorbidity was hypertension with nearly 68% in both groups followed by diabetes with about 30%. Due to serious health complaints of the study patients, the intake of a large number of drugs was documented. A mean number of taken drugs of 3.6 for all patients and 4.7 for the population at risk were observed. Minimal differences between the populations were found in relation to the place of residence of the patients. A slightly higher proportion of patients coming from Germany were detected for the population at risk (90.9%) compared to the total study population (86.5%).

### Frequency of potential drug-drug interactions

The analysis showed at least one PDDI for 296 patients (Table 2). This corresponds to 56.5% of the total population and 77.3% of the population at risk. The most frequent PDDI category was moderate with 268 affected patients. Major PDDIs were seen for 17.7% of the total population and 24.3% of the population at risk. Thus, for almost every fourth patient of our analysis who took at least two drugs simultaneously for 80 days the risk of a drug-drug interaction (DDI) exists. The mean number of all PDDIs ranged from 2.3 for all patients to 3.2 for the population at risk to 4.1 for patients with at least one PDDI.

**Table 2:** Frequency of potential drug-drug interactions

	Total		Minor		Moderate		Major	
**Number of patients with potential drug-drug interactions (total number of patients / percentage of patients)**								
	**n**	**%**	**n**	**%**	**n**	**%**	**n**	**%**
All patients (*n* = 524)	296	56.5	151	28.8	268	51.1	93	17.7
Patients with ≥2 drugs (*n* = 383)	296	77.3	151	39.4	268	70.0	93	24.3
**Mean number of potential drug-drug interactions**								
All patients (*n* = 524)	2.3		0.5		1.6		0.2	
Patients with ≥2 drugs (n = 383)	3.2		0.7		2.2		0.3	
Patients with potential drug-drug interactions (*n* = 296)	4.1		0.9		2.8		0.4	

### Comparison of patients with or without potential drug-drug interactions

The comparison of patients with or without PDDI provided indications for possible correlating factors (Table 3). The most relevant difference was observed for the place of residence. The proportion of patients with place of residence in Germany was distinctly higher for patients with PDDI (92.9%) than for those without (78.1%). Additionally, the mean number of taken drugs differed considerably between patients with (5.3) and without (1.5) PDDI. Regarding the remaining variables of age, sex, race, number of and most frequent comorbidities, no or only marginal differences were found.

**Table 3:** Characteristics of patients with or without potential drug-drug interactions

		Patients without potential drug-drug interactions (*n* = 228)		Patients with potential drug-drug interactions (*n* = 296)	
		n (%) / Mean ± SD		n (%) / Mean ± SD	
Age		64.6	13.2	64.2	12.3
Sex	*Male*	179	(78.5)	232	(78.4)
	*Female*	49	(21.5)	64	(21.6)
Place of residence	*Germany*	178	(78.1)	275	(92.9)
	*Switzerland*	50	(21.9)	21	(7.1)
Race	*White*	214	(93.9)	287	(97.0)
	*Black*	3	(1.3)	1	(0.3)
	*Asian*	2	(0.9)	4	(1.4)
	*Unknown*	9	(3.9)	4	(1.4)
Number of comorbidities	*0*	43	(18.9)	65	(22.0)
	*1*	84	(36.8)	110	(37.2)
	*2*	69	(30.3)	74	(25.0)
	*3*	29	(12.7)	37	(12.5)
	*4*	3	(1.3)	8	(2.7)
	*5*	0	(0)	2	(0.7)
Most frequent comorbidities	*Hypertension*	161	(70.6)	198	(66.9)
	*Diabetes*	64	(28.1)	92	(31.1)
	*Stroke/TIA*	25	(11.0)	34	(11.5)
	*COPD*	21	(9.2)	32	(10.8)
	*Peripheral arterial disease*	19	(8.3)	26	(8.8)
Number of taken drugs		1.5	1.3	5.3	2.1

*SD* Standard deviation, *TIA* Transient ischaemic attack, *COPD* Chronic obstructive pulmonary disease

### Comparison by country

**Fig. 1:**
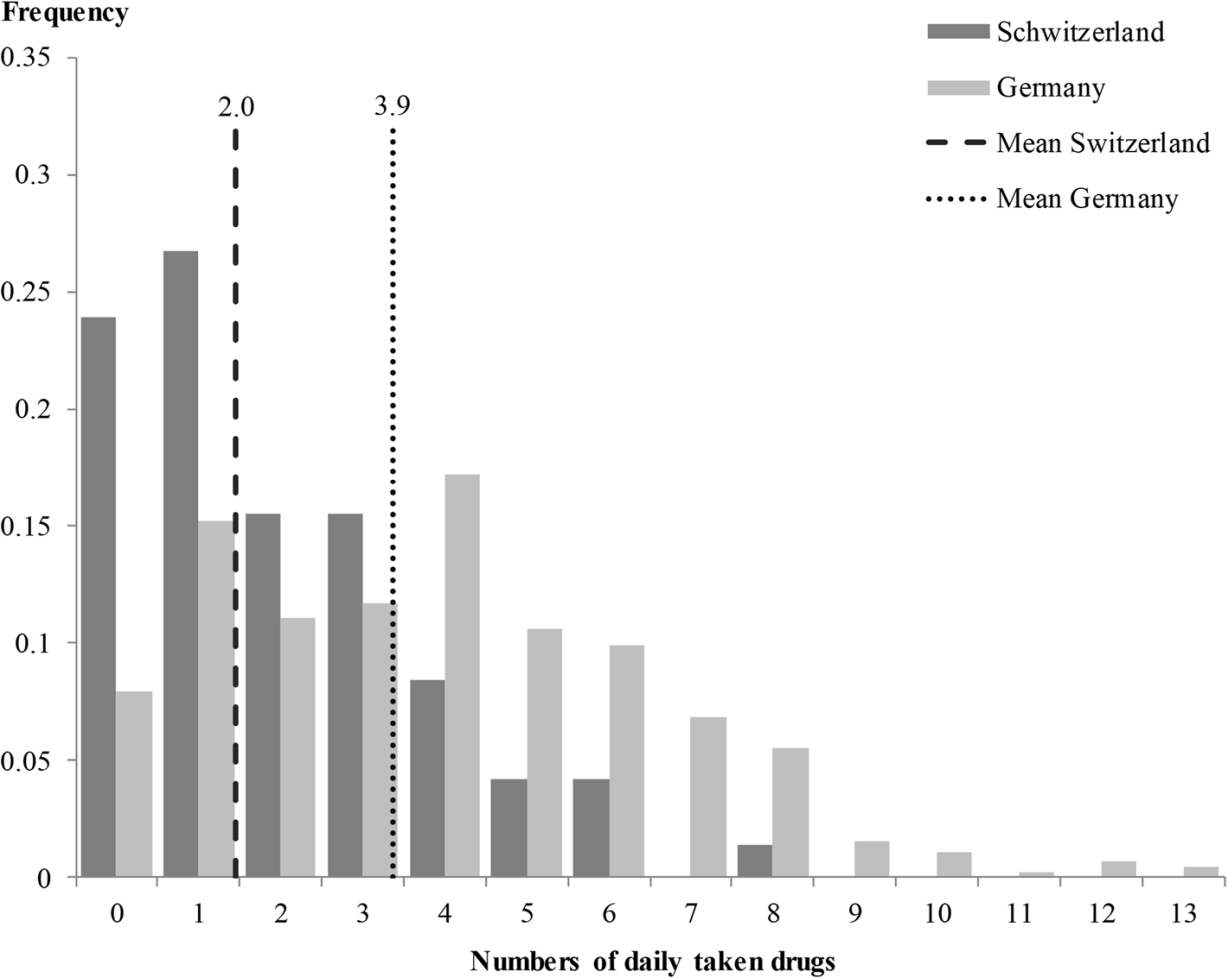
Proportion of patients and number of daily taken drugs in Germany and Switzerland

The high differences shown previously regarding the place of residence of patients with or without PDDI have led to further analyses of patients from Germany compared to those from Switzerland. For the total study population, frequency of all PDDI was twice as high in Germany as in Switzerland. Table 4 shows that 60.7% of all German patients and 29.6% of all patients from Switzerland had a PDDI independent from its category. The difference decreased for the population at risk but was still 19 percentage points. These variations of PDDI frequency in the different places of residence appeared to be significant according to the chi square test. For the total study population, associations with the place of residence were found for every PDDI category. Concerning the population at risk, correlations were observed for moderate and major PDDI. Only the frequency of minor PDDI was not significantly related to the place of residence. The differences in the frequency of PDDIs between both countries may be a consequence of differences in the absolute number of simultaneous taken drugs. Figure 1 shows that patients from Switzerland took less drugs simultaneously compared to the German patients. The average number of daily taken drugs differed between patients from Germany and Switzerland from 3.9 to 2.0. These differences were not alone explainable by the morbidity level, which was widely comparable between the patient groups (data not shown).

**Table 4:** Association between potential drug-drug interactions and place of residence by chi^2^-test

	Potential drug-drug interactions											
	Total			Minor			Moderate			Major		
	n	%	*p*-value	n	%	*p*-value	n	%	*p*-value	n	%	*p*-value
**All patients (*n*** **= 524)**												
Place of residence												
*Germany*	275	60.7		139	30.7		250	55.2		90	19.9	
*Switzerland*	21	29.6	< 0.001	12	16.9	0.017	18	25.4	< 0.001	3	4.2	0.001
**Patients with ≥ 2 drugs (*n*** **= 383)**												
Place of residence												
*Germany*	275	79.0		139	39.9		250	71.8		90	25.9	
*Switzerland*	21	60.0	0.010	12	34.3	0.514	18	51.4	0.012	3	8.6	0.023

## Discussion

Our analysis yielded high proportions of patients with a minor, moderate or major PDDI. Any kind of PDDI was found for just over half of all patients and just over three quarters of patients at risk. The moderate PDDI category was the most frequent one. Patients with and without PDDI differed particularly concerning their place of residence, with higher proportions of PDDI in Germany. In addition, the average number of daily taken drugs varied considerably between Germany and Switzerland.

However, our results are also subject to some limitations. First, we have a very special and highly selected patient clientele. Therefore, the transferability of these results is limited. In addition, our analysis does not allow us to make any statements as to whether any interactions have actually occurred in patients with PDDI. Moreover, this analysis was not intended to denounce physicians in any way, but to draw attention to the problems of polypharmacy and PDDI and to sensitize people to the problem. In addition to possible interactions, polypharmacy also harbors other dangers. There could be cumulative ADEs as well as a mutual intensification of the effect of the drugs (e.g. the simultaneous use of two blood-thinning drugs) [20].

Nonetheless, we are aware that in some cases polypharmacy is intentionally accepted, as international studies showed positive effects and the non-administration of drugs could have even more serious consequences [21]. In addition, it is shown that some potential interactions are only relevant for patients with a certain co-morbidity. We did not check whether this was the case in our population. It should also be noted that, for some patients, the baseline data was several years old. Therefore, it would be conceivable that certain interactions were not known at that time, but are now listed in the interaction database. In addition, it must be taken into account that we used an US-American database because of the accessibility. In comparison to German or European ones, it is possible that other PDDIs are listed in the database of drugs.com. A reason for this could be a varying population composition regarding age or race where active agents work differently. In addition, our assumption that a daily intake of at least two drugs equals a concurrent intake is to scrutinize since we are not able to differentiate the time of intake (e.g. morning, evening). This resulting uncertainty of whether drug-drug interactions are an issue and some other limitations listed may lead us to overestimate the actual proportion of patients with interactions.

The international literature regarding PDDI is quite heterogeneous. Guthrie and colleagues reported a frequency of potentially serious DDI in 13.1% of Scottish adults in 2010 [22]. This number is rather similar to the frequency of major PDDI (17.7%), but distinctly lower than the value for any PDDI (56.5%) in our analyzed population. However, Johnell and Klarin described frequencies of potentially clinically relevant DDIs in 26.0% and of potentially serious DDIs in 5.0% of elderly Swedish people in 2005 [23]. A Swiss study from 2010 observed a proportion of 40.0% in a cohort of HIV-infected persons [24]. One German prospective study from 2007 analyzed patient records of an internal hospital ward and found a proportion of 68% in patients with at least one interacting drug combination [16]. This result is approximately comparable to our overall results of 56.5% for all patients and 77.3% for patients with more than two drugs respectively. Nevertheless, a direct comparison between the results of our present analysis and the international published numbers remains difficult. This may be due to the specific characteristics of our study population, differences in health care systems or varying study designs and assumptions. Literature concerning the variations in the frequency of PDDI between Germany and Switzerland could actually not be found.

Regarding possible explanations for the variations between these two countries, differences in the average number of daily taken drugs between Swiss and German patients were already been mentioned, but certain other reasons would be possible. First, unlike Germany, Switzerland has a so-called positive list of drugs [25, 26]. Such positive list (or formulary) defines a list of drugs reimbursed by a third party payer (e.g. health insurance). Thus, presumably the number of reimbursable drugs differs distinctly between these two countries. Moreover, according to the Federal Institute for Drugs and Medical Devices, there are 102,754 marketable medicinal products in Germany [2]. The Swiss Agency for Therapeutic Products reports only 8259 authorized medicinal products [27]. These differences combined with a fragmented health care system with a free choice and high number of physicians may increase the risk for PDDIs. In particular, patients with chronic diseases often have a large number of different health care providers who may prescribe drugs without knowing of already taken medications. In Germany, the pharmacists should actually check whether there are interactions (probably not systematic) between prescribed drugs. However, they only see the drugs on the specific prescription and know nothing about already taken medications. Overall it seems to be indicated to take measures that lead to improved information transparency for drug prescriber, pharmacists and patients (e.g. digital patient files). Bergk and colleagues already developed suggestions concerning more access for general practitioners or specialists to drug interaction information systems in 2004 [28]. Such systems could give information on PDDI, their clinical relevance, but also patient characteristics and management recommendations based on them [29].

## Conclusions

In this highly selected patient population, a reasonably large proportion of PDDIs was observed. The rates varied between the three categories with highest values for moderate PDDIs. Largest descriptive differences in patients with or without any PDDI were found regarding their place of residence. The presented results are intended to raise awareness of the risks of polypharmacy and PDDIs. Especially for older patients with a high number of comorbidities and prescribed medications, it is important to keep this problem in mind. Adequate cooperation between the various health care providers and new technologies in the health care sector can help to minimize these risks.

## Data Availability

The datasets used and/or analyzed during the current study are available from the corresponding author on reasonable request.

## Abbreviations

PIM: Potentially inappropriate medication
ADE: Adverse drug events
PDDI: Potential drug-drug interaction
ICD: Implantable cardioverter defibrillator
DDI: Drug-drug interaction
